# An analysis of the waning effect of COVID-19 vaccinations

**DOI:** 10.5808/gi.23088

**Published:** 2023-12-29

**Authors:** Bogyeom Lee, Hanbyul Song, Catherine Apio, Kyulhee Han, Jiwon Park, Zhe Liu, Hu Xuwen, Taesung Park

**Affiliations:** 1 Department of Industrial Engineering, Seoul National University, Seoul 08826, Korea; 2 Interdisciplinary Program of Bioinformatics, Seoul National University, Seoul 08826, Korea; 3 Department of Statistics, Seoul National University, Seoul 08826, Korea

**Keywords:** booster doses, COVID-19, immunity, vaccination, waning effect

## Abstract

Vaccine development is one of the key efforts to control the spread of coronavirus disease 2019 (COVID-19). However, it has become apparent that the immunity acquired through vaccination is not permanent, known as the waning effect. Therefore, monitoring the proportion of the population with immunity is essential to improve the forecasting of future waves of the pandemic. Despite this, the impact of the waning effect on forecasting accuracies has not been extensively studied. We proposed a method for the estimation of the effective immunity (EI) rate which represents the waning effect by integrating the second and booster doses of COVID-19 vaccines. The EI rate, with different periods to the onset of the waning effect, was incorporated into three statistical models and two machine learning models. Stringency Index, Omicron variant BA.5 rate (BA.5 rate), booster shot rate (BSR), and the EI rate were used as covariates and the best covariate combination was selected using prediction error. Among the prediction results, Generalized Additive Model showed the best improvement (decreasing 86% test error) with the EI rate. Furthermore, we confirmed that South Korea’s decision to recommend booster shots after 90 days is reasonable since the waning effect onsets 90 days after the last dose of vaccine which improves the prediction of confirmed cases and deaths. Substituting BSR with EI rate in statistical models not only results in better predictions but also makes it possible to forecast a potential wave and help the local community react proactively to a rapid increase in confirmed cases.

## Introduction

The coronavirus disease 2019 (COVID-19) pandemic, caused by the severe acute respiratory syndrome coronavirus 2 (SARS-CoV-2), has had a devastating impact on human health and economic activities around the globe [1]. The virus, which first emerged in late 2019, quickly spread around the world to become a global pandemic, with cases reported in all corners of the world. As of early 2023, the virus has infected over 650 million people and caused over 6 million deaths, making it one of the deadliest pandemics in human history [2].

Governments around the world implemented various measures to curb the spread of the virus and protect the general public health. These policies included travel bans, quarantine protocols, closures of educational institutions, and social distancing measures. To evaluate the efficacy of these lockdown measures, a metric known as the Stringency Index (SI) has been employed [3]. The SI quantifies the degree of strictness of these measures and has been utilized to monitor the effectiveness of the implemented policies in controlling the spread of the virus, as well as to make predictions about the trajectory of the pandemic.

In the ongoing effort to combat the COVID-19 pandemic, the emergence of new variants of the virus also presented a significant challenge. These variants which arise due to a genetic mutation, have been observed to exhibit increased transmissibility, altered disease severity, morbidity, and reduced sensitivity to vaccines, raising concerns about their potential impact on the pandemic [4]. One such variant of concern is the Omicron variant (B.1.1.529), which first emerged in November 2021 and has since spread rapidly to multiple countries [5]. Among omicron's subvariants, BA.5 has been the most dominant of all the strains, in many countries worldwide until late 2022 [6].

In addition to the emergence of these variants, the phenomenon of the "waning effect" or "vaccine fade" has been recognized as a contributing factor to the transmission dynamics of COVID-19 [7]. The waning effect refers to a decline in the level of immunity provided by a vaccine over time, which can occur due to a variety of factors such as the decline of antibody concentrations in the body, loss of immune memory, and the emergence of vaccine-resistant strains. The waning effect can therefore lead to an increased susceptibility to infection and necessitates additional doses for adequate protection. Several studies have been explaining the effect of vaccination in terms of hospitalizations and deaths [8-10] and its effectiveness against the COVID-19 infection, which wanes within a few months of receiving the second dose [11,12]. In a recent study, an additional dose after the second dose restored the vaccine’s effectiveness against COVID-19 [7]. In this study, we will refer to these additional doses as "booster doses," with the designation applying to any doses administered after the second dose.

One of the key challenges in the COVID-19 crisis has been to accurately forecast the spread of the pandemic. Researchers from different fields have contributed to this challenge using various models including statistical models [13-19], machine learning models [20-25], and mathematical models [26-30]. In this study, we evaluated the impact of the waning effect measured using the effective immunity (EI) rate variable, in forecasting the future spread of the SARS-CoV-2 virus in Korea. We believe that the EI rate is a good measure for observing the waning effect, in that the EI rate may decrease over time but the cumulative vaccination rate (VR) always increases with time. The aims of the study include (1) to examine the effect of incorporating the EI rate variable on the prediction accuracy of the models, and (2) to determine the approximate onset time of the waning effect. This can be applied in predicting the next waves of the pandemic. This study employs both statistical and machine learning models to analyze the data and test the proposed objectives. The results of this research could provide valuable insights for decision-makers and public health officials in their efforts to control and manage the spread of COVID-19.

## Methods

### Response variables

The COVID-19 data consists of daily series of confirmed cases, death cases, intensive care unit (ICU) patients, VRs according to the number of inoculations (per hundred people), and the SI of South Korea. All variables were downloaded from *Our World in Data* (OWID) [31]. The daily confirmed cases and deaths were officially collected through the Center for Systems Science and Engineering (CSSE) at Johns Hopkins University [32], while the ICU patient data was officially collected by the OWID team. Missing dates of confirmed cases, deaths, and ICU patients in OWID were downloaded from Korea's COVID-19 dashboard [33]. We used daily confirmed cases, deaths, and ICU patient data from South Korea. We used both raw and smoothed data (a 7-day window is applied to smooth the data). Our train period is from January 1, 2022 to October 24, 2022. Our test period is from October 25, 2022 to November 7, 2022 (14 days). This period was chosen due to the high proportion of daily cases caused by the Omicron variants.

### EI rate

**Fig. 1.**
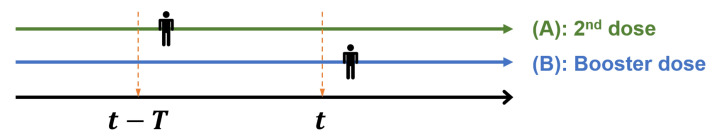
The definition of effective immunity.

The EI rate is defined for each time point in our analysis and is an integrated measure for the second and booster doses. Being infected also creates a natural immunity in people but the individual data to distinguish whether an individual is infected or not is currently unavailable. Although infection also leads to natural immunity, data to distinguish individual infection status is currently unavailable.


$$EI_{t}=\frac{V_{2t}\;+\;V_{3t}}{\text{Total population,}}$$

where *t* is any specific date, and *V*_2*t*_ and *V*_3*t*_ are the numbers of people who received the second and booster doses of the vaccines, respectively, during the time interval [*t*-*T*,*t*]. Here, *T* indicates the length of days an individual can retain his/her immunity (effective period or effective days) obtained from the second or booster dose before the waning effect of vaccination starts. In other words, it is assumed that the waning effect starts *T* days after vaccination, regardless of the date of observation. *T* varies in our study in order to observe varying prediction errors for each value of *T* of vaccination. According to the literature [34], this is usually after 90 days but it may vary with the country and type of COVID-19 vaccine received. Therefore, in our study, candidates for *T* were selected as 30, 60, 90, and 120 days. In South Korea, the booster dose was inoculated approximately three months after the second dose following the government policy, considering a time point *t* when a person received the first dose of vaccine. Then, we can safely assume there does not exist the same individual in the time interval [*t*-*T*,*t* ], as long as *T* ≤90 days. This is because an individual receives the next dose 90 days after the previous dose. Fig. 1 shows how *V*_2*t*_ and *V*_3*t*_ are counted for any specific date *t*.

Suppose an individual received her second dose in the time interval [*t*-*T*,*t*], the individual will receive the next dose after 90 days. If *T*≤90, then she will be counted only once (in *V*_2*t*_ and not in *V*_3*t*_). Otherwise, if *T*>90, it is possible she is counted twice (both in *V*_2*t*_ and *V*_3*t*_). Thus, if we select any *T*, an individual cannot receive two doses at that time interval. For example, at *T* = 120, each individual with immunity may appear twice after receiving the next dose 90 days after the previous dose and included in *V*_2*t*_ and *V*_3*t*_ (the EI rate is taken as 1.0 in this case). Therefore, the proportion of people with immunity may exceed 1.0, which is unreasonable. In summary, the EI has the advantage that we can simply estimate the proportion of the whole population who has immunity at any given time point without individual data.

### Covariates and lagging effects

The covariates considered in this study include the government SI, Omicron variant BA.5 rate, booster shot rate (BSR), and the EI rate. The SI was obtained from the Oxford COVID-19 Government Response Tracker [35], BSR from OWID [36], and the proportion of the Omicron variant BA.5 was downloaded from the CoVariants website [37] and GISAID [38-40]. The list of covariates is summarized in the table (Table 1).

Considering a given response variable as *Y_t_* and covariates as *X_t_* = (*x*_1*t*_,…,*x_Kt_*), it is important to note that the effects of vaccination and intervention policies on the spread of COVID-19 may take some time to be observed. Therefore, it would be reasonable to consider this factor when predicting future daily confirmed cases, daily death cases, or ICU patients. We used a total of four lags: 7, 14, 21, and 28 days for SI and BSR in our models as follows: *X*_t-7_ + *X*_t-14_ + *X*_t-21_ + *X*_t-28_. Five different covariate combinations, in addition to the null model (no covariates), were used to predict our response variables. The list of covariate combinations is summarized in the table (Table 2).

### Models

#### AutoRegressive Moving Average Model

AutoRegressive Moving Average (ARMA) models for time series analysis were first suggested in *Time Series Analysis: Forecasting and Control* [41]. Since ARMA models could be applied only to stationary time series, multiplicative seasonal Autoregressive Integrated Moving Average (ARIMA) models were developed to utilize differentiation and include seasonality in ARIMA models [42]. To obtain future predictions, an R package *forecast* was used for fitting ARIMA and seasonal ARIMA models and the principle of parsimony was applied in this analysis. Instead of using the *auto.arima()* function in *R* like in previous studies [43], we compared Akaike information criterion and Bayesian information criterion values [44] for all possible seasonal ARIMA models fitted and chose the best model by limiting the orders of models to integer values chosen beforehand. This prevented the overfitting problem.

#### Generalized Additive Model

The Generalized Additive Model (GAM) is a regression model that allows the learning of nonlinear relationships between each covariate and mean response E(*Y*), using the smooth function *f_i_* (*X_i_*) [45]. Here, we assumed our response variables followed a Poisson distribution and different smoothing functions *f_j_* were used depending on the covariates. For weekdays and dates, cubic splines and P-splines were used, respectively and thin plate regression splines were used for vaccination covariates and SI [46]. R package *mgcv* was used for fitting GAM models [47,48].

#### Time series Poisson

Time series Poisson aims to model the conditional mean *E*(*Y_t_*|*F*_*t*-1_) by a process {*λ_t_*}, such that *E*(*Y_t_*|*F*_*t*-1_) = *λ_t_*. In this study, to consider negative covariate effects, we used a logarithmic link function and the model can be written again as follows:


$$\log\left(\lambda_{t}\right)=\beta_{0}+\sum_{k=1}^{p}\beta_{k}\log\left(Y_{t-k}+1\right)+\sum_{l=1}^{q}\;\alpha_{l}v_{t-l}+\eta^{t}X_{t},$$

where, *F_t_* the history of the joint process {*Y_t_*,*λ_t_*,*X*_*t*+1_} and *η* represents the effects of covariates. We also applied the Poisson assumption for this model, i.e., *Y_t_*|*F*_*t*-1_ ~ Poisson (*λ_t_*). Time Series following Generalized Linear Models (TSGLMs) are introduced in *tscount: An R Package for Analysis of Count Time Series Following Generalized Linear Models* [49].

#### Light Gradient Boosting Machine

Light Gradient Boosting Machine (LightGBM) is a gradient boosting decision tree algorithm that can be used for tasks like regression and classification. LightGBM consists of decision trees as weak learners and adds models into the tree using a greedy style approach [50]. Based on the adaptive boosting algorithm, gradient boosting machines (GBM) can build a strong regression learner by iteratively combining a set of weak regression learners. GBM uses gradient descent for minimizing the loss function of a strong regression learner. To build our lightGBM model, the ‘LightGBM’ package in Python was used [51].

#### Bidirectional long short-term memory network

To deal with time series data, long short-term memory (LSTM) network was considered as the deep learning approach [52]. Since LSTM takes only past information when training, we adopted bidirectional LSTM (Bi-LSTM) to consider backward propagation information as well [53]. The optimal bandwidth of the training period is selected among 7, 14, or 21 which yields the least validation mean squared error. To improve the model performance, the training process was conducted in both forward and backward directions. The model structure considered two hyperparameters: layer number {2, 3} and dropout rate {0, 0.2}. The model was developed in Python version 3.7.6 using Keras (version 2.4.3, https://github.com/keras-team/keras) and TensorFlow (version 2.3.0, https://github.com/tensorflow/tensorflow) libraries.

### Model performance

For a given covariate combination and prediction model, performance was measured using the weighted mean absolute percentage error (WMAPE) that measures a model prediction accuracy using the test data. The model and covariate combination with the smallest test WMAPE values is taken as the best for forecasting. WMAPE is defined as follows:


$$WMAPE=\frac{\sum_{\text{t}=1}^{\text{T}}\left|y_{t}\;–\;\hat{y_{t}}\right|}{\sum_{\text{t}=1}^{\text{T}}\left|y_{t}\;\right|},$$

where *y_t_* and $\hat{y_{t}}$ are actual and predicted values, respectively.

## Results

### EI rate improves COVID-19 prediction accuracy

For the five models (ARIMA, GAM, TSGLM, LightGBM, and Bi-LSTM) the prediction results for the daily confirmed cases for the vaccination lasting period *T* = 90, using raw data are summarized (Table 3). The covariate combination numbers of Table 3 are in the same order with Table 2.

We compared covariate combinations SI + BSR and SI + EI. In the same way, covariate combinations SI + BA.5 rate + BSR and SI + BA.5 rate + EI are compared since the former uses BSR with BA.5 rate and the latter uses EI with BA.5 rate. We observed that using EI improves prediction accuracy for covariate combinations SI + EI and SI + BA.5 rate + EI, in comparison to combinations SI + BSR and SI + BA.5 rate + BSR, respectively. For ARIMA and GAM, prediction accuracy improved for combinations SI + EI and SI + BA.5 rate + EI, whereas for TSGLM, LightGBM, and Bi-LSTM, combination SI + EI showed higher prediction accuracy. Among all models, Bi-LSTM with EI as a covariate showed the best prediction. Results for smoothed data using daily confirmed deaths and ICU patients are listed in Application Note.

### Time to onset of waning effect

To find the approximate vaccination lasting time *T* before the onset of waning effect, we compared WMAPE values of all models using the covariate combinations SI + EI and SI + BA.5 rate + EI with the baseline models (covariate combinations SI + BSR and SI + BA.5 rate + BSR) for various values of T. The test WMAPE values (daily confirmed cases) of combinations SI + EI and SI + BA.5 rate + EI for *T* = 30, 60, 90, and 120 are summarized in Table 4. Note that *T* = 150 is not introduced here since EI exceeds 1.0 (and is considered as 1.0) for the *majority* of the training period, which indicates EI cannot be a good predictor. Meanwhile, *T* = 120 is included in the analysis since there exist periods where EI exceeds 1.0, but not as much as when *T* = 150.

Overall, 90 days performs best for both covariate combinations (SI + EI and SI + BA.5 rate + EI). In order of performance, 90, 30, 60, and 120 are appropriate vaccination lasting times to be applied for the EI rate. The mean WMAPE values for all five models for each type of data are summarized in Table 5. Note that the model average values of Table 4 are in the first column (raw daily cases).

For both covariate combinations SI + EI and SI + BA.5 rate + EI, we observed that 90 days applied to the EI rate best reduces prediction error for raw daily cases and deaths. For raw ICU patients, 60 days showed the best performance. For smoothed data, 30 days showed the best performance for daily cases and deaths. 60 days showed the best performance for daily cases and ICU patients. Finally, 90 days performed well for ICU patients.

## Discussion

The COVID-19 pandemic represents the biggest global shock in decades that affected all major aspects of life [54,55]. Due to a lack of specific therapeutic agents or effective treatment against COVID-19, the outbreak elicited immense global interest in the development and distribution of safe COVID-19 vaccines capable of stopping the spread of COVID-19 disease. The Coalition for Epidemic Preparedness Innovations (CEPI) started working with global health authorities, biotech, governments, and academic collaborators to support the development of vaccines against COVID-19 [56,57]. The COVID-19 vaccine R&D landscape developed at an unprecedented scale and speed in that by December 11, 2020, the U.S. Food and Drug Administration issued the first emergency use authorization for the PfizerBioNTech COVID-19 [58,59]. After that, other countries followed and issued approvals for vaccines like the Moderna vaccine, Oxford-AstraZeneca vaccine, Sputnik V vaccine, and Johnson & Johnson vaccine [60]. The fast development of COVID-19 vaccines was expected to play the game-changer role in fighting the spread of COVID-19.

However, although the vaccines could reduce the severity of COVID-19, they could not stop the spread of the virus permanently [61]. A vaccinated person could still contract the virus or pass the virus to another individual. Furthermore, one dose of the vaccine could not provide lasting immunity. Second doses and booster shots have to be received by the population to maintain immunity against COVID-19. The emergence of SARS-CoV-2 variants like the Omicron variant also posed a challenge to the efficacy of COVID-19 vaccines. A lot of uncertainties were raised over how long the primary vaccination series would remain effective and the ideal timing for booster doses. Several studies provided robust evidence of the waning effect of vaccine immunity over time [7,62,63].

In forecasting future COVID-19 situations, factors such as the waning effect and variants must be considered, thus highlighting the importance of additional doses and government policies. In terms of deciding the optimal time for booster shots before the waning effect occurs rate with only population data in the absence of individual data of vaccinated or infected people. Although studies discovered that immunity obtained by vaccinations may yield more durable protection than natural infection, immunity obtained by being infected still has a significant effect on the duration of immunity levels [64]. If subject-specific vaccination or infection data is available, there will be an improvement in prediction accuracy. Furthermore, the reinfection rate can be considered to estimate EI more accurately and predict potential waves in the future. While our analysis focused only on South Korea, our method of calculating the EI rate is straightforward and can be applied to other countries. This approach could improve the prediction of future pandemic patterns, including cases, deaths, and ICU patients.

When we introduced the EI rate that we defined into each prediction model, the degree of improvement in prediction performance was different. For instance, when predicting raw daily confirmed cases, the GAM showed the most significant increase in performance (test WMAPE decreased from 0.785 to 0.211, an 86% decrease) when utilizing the booster rate. It was the second-best performance following the Bi-LSTM (test WMAPE 0.189). Since these two models can consider the non-linearity between the predictors and response variables, it can be inferred that modeling the nonlinear relationship between EI and response variables may contribute to improved prediction performance.

In general, immunity starts waning after vaccination. To model the waning of immunity, we hypothesized that the population loses EI against the SARS-CoV-2 virus after a certain number of days (T days) from the last vaccination. Our models showed the best performance with an EI duration of *T* = 90 days, which suggests that the immunity waning effect likely starts around 90 days following the last vaccine dose. Thus, although not derived from experiments at an individual level, such as a serological test, we suggest our best-predicted *T* as evidence to estimate the onset of the waning effect. Understanding this timing is beneficial for healthcare policy decisions, such as establishing guidelines for the administration of booster doses.

In conclusion, we can conclude immunity loss from inoculations occurs approximately after three months.Compared to utilizing the original booster shot rate, using the EI rate significantly reduces prediction error for all response variables: confirmed cases, deaths, and ICU patients. Furthermore, even though the most appropriate vaccination lasting time does vary between raw and smoothed data, we have shown that considering 90 days for the South Korean population is a reasonable choice for accurate predictions, especially on confirmed cases and deaths.

## Figures and Tables

**Table 1.** Covariate list

Covariate	Abbreviation	Explanation
Stringency Index	SI	The level of strictness in government policies and interventions in response to pandemics
Omicron variant BA.5 rate	BA.5 rate	The prevalence rate of the BA.5 subvariant of the COVID-19 Omicron variant
Vaccination rates	VR	The percentage of fully vaccinated people or received booster dose in a population
Booster shot rate	BSR	The percentage of people in a population who have received a booster dose of the COVID-19 vaccine
Effective immunity rate	EI rate	The percentage of the people in a population who have received a vaccination within the past T days (e.g., 90 days)

COVID-19, coronavirus disease 2019.

**Table 2.** Covariate combinations

No.	Covariate combination
#1	Null model (no covariates)
#2	SI + BA.5 rate
#3	SI + BSR
#4	SI + EI
#5	SI + BA.5 rate + BSR
#6	SI + BA.5 rate + EI

SI, Stringency Index; BSR, booster shot rate; EI, effective immunity.

**Table 3.** WMAPE values for daily confirmed cases for raw data (T = 90)

Covariate combination	ARIMA	GAM	TSGLM	LightGBM	Bi-LSTM
#1 Null	0.417	1.392	0.648	0.508	0.925
#2 (SI + BA.5 rate)	0.435	0.502	0.383	0.923	0.813
#3 (uses booster rate)	0.409	0.785	1.133	0.636	0.258
#4 (uses EI, compare with #3)	0.268	0.211	0.379	0.634	0.189
#5 (uses BA.5 rate)	0.419	1.589	1.175	0.866	0.265
#6 (uses EI, compare with #5)	0.336	0.222	1.272	0.895	1.090

WMAPE, weighted mean absolute percentage error; ARIMA, Autoregressive Integrated Moving Average; GAM, Generalized Additive Model; TSGLM, Time Series following Generalized Linear Model; LightGBM, Light Gradient Boosting Machine; Bi-LSTM, bidirectional long short-term memory; SI, Stringency Index; EI, effective immunity.

**Table 4.** Test WMAPE values for variable combinations SI + EI and SI + BA.5 rate + EI for daily confirmed cases.

Vaccination lasting time (*T*)	Covariate combination	ARIMA	GAM	TSGLM	LightGBM	Bi-LSTM	Model Average
30	SI + EI	0.366	0.202	0.384	0.834	0.310	0.419
	SI+BA.5 rate + EI	0.441	0.216	0.372	0.340	1.330	0.540
60	SI + EI	0.300	0.202	0.374	0.511	1.205	0.518
	SI+BA.5 rate + EI	0.380	0.463	0.520	0.340	1.232	0.587
90	SI + EI	0.268	0.202	0.379	0.547	0.333	0.346
	SI+BA.5 rate + EI	0.336	0.222	0.480	0.340	0.625	0.401
120	SI + EI	0.431	0.202	0.408	1.088	1.044	0.635
	SI+BA.5 rate + EI	0.569	0.682	0.715	0.340	1.809	0.823

WMAPE, weighted mean absolute percentage error; SI, Stringency Index; EI, effective immunity; ARIMA, Autoregressive Integrated Moving Average; GAM, Generalized Additive Model; TSGLM, Time Series following Generalized Linear Model; LightGBM, Light Gradient Boosting Machine; Bi-LSTM, bidirectional long short-term memory.

**Table 5.** Mean WMAPE values for five models and variable combinations SI + EI and SI + BA.5 rate + EI.

Vaccination lasting time (*T*)	Covariate combination	Raw			Smoothed		
		Daily cases	Daily deaths	ICU patients	Daily cases	Daily deaths	ICU patients
30	SI + EI	0.419	0.963	0.09	0.130	0.094	0.074
	SI + BA.5 rate + EI	0.540	0.524	0.097	0.158	0.073	0.104
60	SI + EI	0.518	0.642	0.045	0.120	0.096	0.071
	SI + BA.5 rate + EI	0.587	0.545	0.063	0.229	0.185	0.034
90	SI + EI	0.346	0.419	0.100	0.124	0.097	0.055
	SI + BA.5 rate + EI	0.401	0.496	0.073	0.199	0.091	0.087
120	SI + EI	0.635	0.511	0.108	0.122	0.176	0.068
	SI + BA.5 rate + EI	0.823	0.535	0.104	0.278	0.209	0.121
Best *T*	SI + EI	90	90	60	60	30	90
	SI + BA.5 rate + EI	90	90	60	30	30	60

WMAPE, weighted mean absolute percentage error; SI, Stringency Index; EI, effective immunity; ICU, intensive care unit.
